# The Elemental Composition of *Fimbristylis ochotensis* in Relation to Its Geothermal Habitat

**DOI:** 10.3390/plants15182773

**Published:** 2026-09-10

**Authors:** Inga Zinicovscaia, Olga Chernyagina, Pavel Nekhoroshkov, Margarita Shvetsova, Nikita Yushin, Octavian G. Duliu, Dmitrii Grozdov

**Affiliations:** 1 Joint Institute for Nuclear Research, Joliot-Curie 6, 141980 Dubna, Russia; p.nekhoroshkov@gmail.com (P.N.);; 2 Horia Hulubei National Institute for R&D in Physics and Nuclear Engineering, 30 Reactorului Str., 077125 Magurele, Romania; 3 Laboratory of the Plants Ecology, Vitus Bering Kamchatka State University, Pogranichnaya St. 4, 683000 Petropavlovsk-Kamchatsky, Russia; 4 Department of Structure of Matter, Earth and Atmospheric Physics, Astrophysics, Faculty of Physics, University of Bucharest, 405, Atomistilor Str., Ilfov, 077125 Magurele, Romania; 5 Geological Institute of Romania, 1, Caransebes Str., 012271 Bucharest, Romania

**Keywords:** *Fimbristylis ochotensis*, neutron activation analysis, elemental composition, transfer factor, enrichment factor, hot springs

## Abstract

*Fimbristylis ochotensis* (Meinsh.) Kom. is a rare and vulnerable plant species of significant scientific and ecological importance. It is an obligate thermophyte that grows exclusively around hot springs and geothermal fields on the Kamchatka Peninsula and in northern Japan. For the first time, the elemental composition of *Fimbristylis ochotensis* and its associated soils was determined using neutron activation analysis, supplemented with ICP-OES and a direct mercury analyzer, on samples collected from three hot spring zones on the Kamchatka Peninsula. In total, 43 elements were quantified in soil samples and 31 elements in plant samples. Both soil and plant material collected near different hot springs exhibited significant variation in elemental composition. Mass fractions of most elements were higher in soil samples, with the exceptions of Zn, Br, Rb, Cs, and Cd. Enrichment factor values indicated moderate to extreme soil enrichment in As, Br, Cd, Sb, and W, while transfer factors greater than unity were obtained for K, Rb, Cs, Br, Zn, S, and Cd, suggesting efficient accumulation of these elements by the plant from the soil.

## 1. Introduction

Thermal habitats represent unique geothermal ecosystems in which extreme physicochemical conditions coincide with unexpectedly rich microbial and plant diversity [1]. These thermal environments, including geysers, fumaroles, and hot springs themselves, are typically classified as extreme habitats owing to their elevated soil mineralization, high concentrations of potentially toxic elements (PTEs), and chronic thermal stress [2,3]. Although bacterial [4,5] and algal communities [6,7,8] inhabiting these systems have received considerable research attention, plants capable of surviving and reproducing under the severe physicochemical constraints of surface geothermal areas remain comparatively understudied [2,3]. This knowledge gap is particularly significant given that plant species able to tolerate extreme conditions have long been recognized as valuable models for ecological and physiological research [2]. Moreover, many of these plant species are not only ecologically specialized or locally endemic but also have medicinal properties [9,10].

Early research on extremophilic plants was summarized in the Stout and Al-Niemi (2002) study [2]. Among more recent contributions, Pantoja-Irys et al. [3] examined plant diversity across five hot springs with contrasting geochemical characteristics in northeastern Mexico. Concurrently, numerous studies have focused on bryophyte species, investigating not only their biodiversity but also their capacity to accumulate PTEs, including Hg, As, and Sb, given that water and soils in geothermal areas often exhibit anomalously high background concentrations of such elements [11,12,13]. To the best of our knowledge, however, no information is currently available regarding the elemental composition of vascular plants growing in thermal zones.

The Kamchatka Peninsula, characterized by intense volcanic activity and a high concentration of thermal springs, represents a particularly promising region for investigating plant communities associated with geothermal habitats [8]. Among the flora of this region of interest is *Fimbristylis ochotensis* (Meinsh.) Kom., a characteristic and persistent companion of thermal anomalies. 

*Fimbristylis ochotensis* (Meinsh.) Kom. (Cyperaceae) (*F. ochotensis*) is an herbaceous annual that forms dense mats of numerous shoots. The stems are 5–40 cm tall, ribbed, with scattered long hairs beneath the inflorescence and brownish sheaths at the base. The leaves are 1–2.5 mm wide, flat, with slightly recurved margins, and are shorter than the stem, though occasionally almost as long. The inflorescence is an umbel-shaped panicle of 2–4 spikelets, with 1–3 bracts, the lowermost of which reaches 2–4 cm in length. 

*F. ochotensis* is an obligate thermophyte, most often found growing alongside hot springs. It grows in dry thermal areas or forms dense borders at the water’s edge. This species forms extensive borders along the banks of hot springs and exhibits remarkable tolerance, surviving in close proximity to the epicenters of thermal fields where soil temperatures in the root zone can reach up to 50 °C [14]. Approximately 27 isolated populations are known in Kamchatka, occurring at altitudes of up to 900 m above sea level [15]. In addition to the Kamchatka Peninsula, the species is also recorded in northern Japan, where it grows under similar conditions [16]. Several adaptive traits contribute to the species’ survival in extreme conditions: a C_4_-type photosynthetic pathway [17], high heat resistance of the cytoplasm in leaf epidermal cells, and symbiotic relationships with soil fungi [18]. 

It was previously shown that *Fimbristylis* species can tolerate high metal concentrations when growing under very acidic conditions. For instance, *Fimbristylis dichotoma* subsp. *podocarpa* (Nees) T.Koyama, which occurs in the solfatara fields of Kyushu, western Japan, has been found to tolerate high concentrations of Al at acidic pH values [19]. Other *Fimbristylis* species growing on non-metalliferous soils have demonstrated a high phytoremediation capacity for potentially toxic elements such as Pb [20], Cr [21], and Cu, Zn, and Cd [22]. At the same time, to the best of our knowledge, no information is currently available regarding the elemental composition of *Fimbristylis ochotensis* or other *Fimbristylis* species growing in thermal zones.

For the first time, three analytical techniques—neutron activation analysis (NAA), inductively coupled plasma optical emission spectrometry (OES), and direct mercury analysis—were employed to determine the mass fractions of chemical elements in *Fimbristylis ochotensis* plants and the corresponding soil samples collected near three hot springs, namely Bolshie Bannye Klyuchi, Malye Bannye Klyuchi and Dachnye Hot Springs on the Kamchatka Peninsula.

## 2. Results and Discussion

### 2.1. Soil Elemental Composition

Thermal anomalies create specific endothermic soils, whose elemental composition depends on the type of host rock involved in the water–rock reaction, the input of magmatic fluids, and the degree of hydrothermal alteration of the parent material. Variations in the mineral composition of hydrothermally altered soils are further controlled by the intensity and duration of hydrothermal activity [23,24]. By combining three analytical techniques, the mass fractions of 43 elements were determined in the analyzed soil samples (Table 1). Among the elements detected, Ni was found only in soils from site 3 (Malye Bannye Klyuchi), while Au and Ga were detected exclusively in the soil from site 4 (Dachnye Hot Springs). A comparison of the elemental mass fractions with those of the upper continental crust revealed that the analyzed samples are enriched in Mg, As, Br, Sb, Cs, W, S, Cu, Cd, Pb, and Hg. Similar enrichment patterns have been observed in other high-temperature geothermal fields, such as Yangbajing in Tibet and Rehai in Yunnan [25], as well as in hydrothermal fields in the Valley of Geysers on the Kamchatka Peninsula [26]. Multielement geochemical anomalies, including Hg, As, Sb, Bi, B, Be, Li, Rb, Cs, W, Sn, Pb, Zn, Mn, Ni and Co, have also been documented above the geothermal field in the Zhangzhou basin in Fujian Province [25]. 

**Figure 1:**
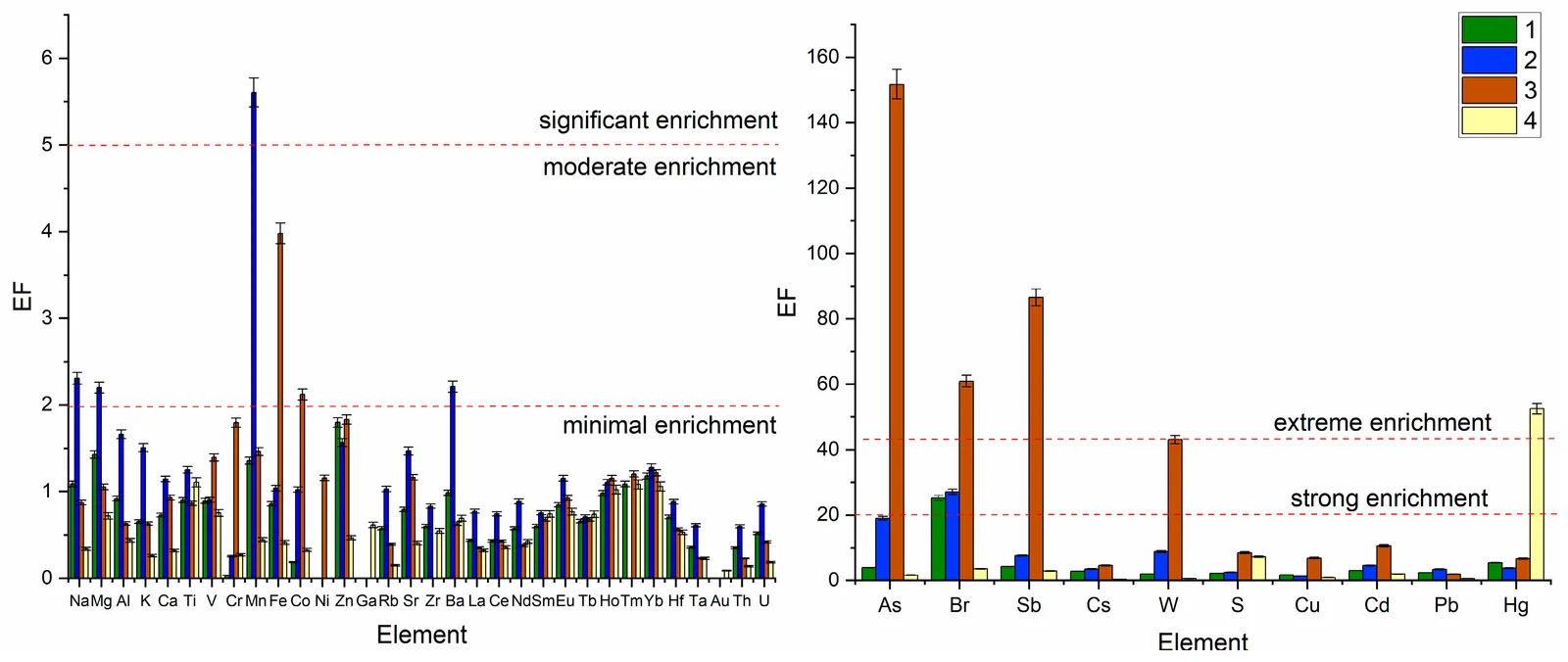
Enrichment factor values for soil samples collected at hot springs of Kamchatka Peninsula (error bars indicate standard deviation).

Enrichment factor (EF), a parameter used to quantify the enrichment of an element in an environmental sample relative to a background, showed minimal soil enrichment in Na, Mg, Al, K, Ca, Ti, V, Cr, Mn, Fe, Co, Ni, Zn, Ga, Rb, Sr, Zr, Ba, La, Ce, Nd, Sm, Eu, Tb, Ho, Tm, Yb, Hf, Ta, Au, Th, and U (Figure 1). At site 1 (Bolshie Bannye Klyuchi), soils were minimally enriched in As, Sb, Cs, W, S, Cu, Cd, and Pb, moderately enriched in Hg, and strongly enriched in Br. At site 2, located within the same hot spring area, the soil showed significant enrichment in As, Sb, and W, and strong enrichment in Br. Soil from Malye Bannye Klyuchi exhibited significant enrichment in Cd and extreme enrichment in As, Br, Sb, and W. At Dachnye Hot Springs, soils were strongly enriched in Hg. 

Hg, As and Sb are considered the best geothermal indicators. These elements tend to decrease in concentration with decreasing water temperature in the geothermal zone [25]. Results of the present study confirm this observation. The highest levels of As and Hg were detected in soils collected near springs with water temperatures of 70 °C (sites 3 and 4). The As, Br, Sb, W and Hg anomalies observed in endothermic soils result from underground thermal waters [24,25,28,29]. Previous studies have shown that As, Sb, and W are enriched in hot spring sediments with high Fe mass fractions [30,31], while Cs is strongly retained by carbonate minerals [31]. The highest mass fractions of As, W, and Sb were determined in the soil from Malye Bannye Klyuchi, which was also characterized by the highest Fe mass fraction (Table 1), whereas high Cs mass fractions in soils correlated with high Ca levels. The Pearson correlation coefficient values between As, Sb, W, Zn, Cu, and Cd ranged from 0.98 to 1.0, indicating strong positive associations among these elements. Together with the elevated Fe mass fractions observed in the same samples, these associations may suggest a common source or co-occurrence with Fe-bearing mineral phases. However, correlation analysis alone cannot establish adsorption onto iron minerals, and this interpretation would require confirmation through mineralogical or chemical speciation analyses.

It is important to mention that the analyzed soils differed significantly in their elemental composition. Distinct site-specific patterns were observed in the distribution of major and trace elements. Thus, the highest mass fractions of Na, Mg, Al, K, Ca, Zn, Rb, Th, U, and Pb were determined in soil collected at site 1 (Bolshie Bannye Klyuchi), suggesting a relatively strong contribution of lithogenic and mineral-derived components at this site. Soil collected in the same area (site 2) was characterized by the highest content of Mn and Ba, indicating a distinct geochemical pattern compared with site 1, potentially associated with differences in mineral composition or local hydrothermal inputs. In contrast, soils collected at site 3 (Malye Bannye Klyuchi) exhibited the highest mass fractions of Cr, Co, Fe, Ni, As, Br, Sr, Sb, Cs, W, Cu, and Cd. The co-enrichment of Fe, As, Sb, W, and several transition metals at this site may indicate a stronger influence of hydrothermal mineralization and/or the accumulation of elements associated with Fe-bearing mineral phases. The elevated Br and Cs mass fractions may additionally reflect differences in the mobility and retention of these elements under local hydrothermal conditions.

The mass fractions of other elements were maximal in soils collected at site 4 (Dachnye Hot Springs). Soils from the Bolshie Bannye Klyuchi area showed a statistically higher mass fractions of Na, Mg, Al, and K (*p* < 0.05) compared with those in the other hot spring areas. These elements mainly originate from the weathering and dissolution of silicate and carbonate minerals [32]. It should be mentioned that in the soil samples (site 1 and 2) collected in the Bolshie Bannye Klyuchi area, the differences in the mass fractions of Sc, Ti, V, Mn, Fe, Zn, As, Br, Zr, Cs, Sm, Eu, Tb, Ho, Yb, S, and Hg were statistically significant (*p* < 0.05). Trace element mass fractions in soil of geothermal areas differ significantly depending on the local parent rock geology, spring water pH, temperature, and evaporation rates [33]. The diversity of geological processes and element retention by soil are the main factors affecting elemental mass fractions [31,32,34].

### 2.2. Fimbristylis Ochotensis Plants Elemental Composition

Acid soils can influence plants through chemical reactions such as increasing the mass fractions of potentially toxic elements (PTEs) or decreasing the availability of nutrients [19]. The mass fractions, as well as the number of elements determined *in F. ochotensis* plants, were lower than those in soils. Specifically, the mass fractions of Ni, Ga, Zr, Ce, Nd, Eu, Ho, Tm, Yb, Hf, Ta, W, and U were below the detection limit of NAA in all analyzed plants. The transfer of elements from soil to plants is controlled by their concentrations and speciation in the soil, as well as by soil physicochemical properties. The absence of certain elements in plants can be explained by their low mobility, the formation of insoluble complexes, and/or exclusion by root cell membranes [35,36]. At the same time, the mass fractions of K, Zn, Br, Rb, Cs, and Cd were higher in plants than in the soil. This pattern may reflect the ability of plants to accumulate elements from atmospheric deposition, geothermal emissions, and precipitation, in addition to soil-derived inputs. In volcanic environments, K, Rb, and Cs act as highly incompatible alkali metals that partition heavily into magmatic gases and geothermal fluids. When these elements reach the surface via fumaroles, they undergo a distinct cycle involving mineral crystallization, water-rock leaching, and uptake by specialized pioneer plant communities [37,38]. The elevated Br levels may be associated with atmospheric deposition of volatile halogen compounds released from fumarolic sources [39]. Thus, the plant-to-soil differences indicate that their elemental composition integrates multiple environmental sources and uptake processes.

According to the results presented in Table 2, both plant and soil samples differed significantly in their elemental composition. 

In general, the highest mass fractions of elements corresponded to their highest levels in the soil. All 31 elements were determined in *F. ochotensis* from site 4 only. This can be associated with the acid soil pH of 3.66, which causes PTEs to desorb from soil particles and dissolve into bioavailable free ionic forms. This drastically increases metal uptake by plants [40]. In plants collected at site 1, the mass fractions of Ti, Cr, Sb, Tb, Au, and Th were below the NAA detection limit. In plants collected from sites 2 and 3, the same pattern was observed; additionally, the mass fractions of V and La were below the detection limit in samples from site 2, and those of V, La, Ba, and Sc were below the detection limit in samples from site 3.

Among the determined elements, the mass fractions of K, Ca, S, and Mg were the highest in all plants. These elements are essential plant macronutrients that participate in various physiological and biochemical processes, including protein synthesis, enzyme activation, turgor pressure regulation, and water transport. They affect plant growth and yield formation [41,42]. The mass fractions of K were higher than those reported for the “Reference Plant” introduced by Markert [40]. In geothermal areas, K, Na, and Ca are the major cations, while Cl, S, and Br are the main anions [43,44]. This can explain the higher mass fractions of Na, Cl, and Br in the analyzed plants compared to the RP. The natural Br content of plants generally does not exceed about 40 ppm; however, in geothermal areas, its content can reach up to 2000 mg/kg [45]. Chlorine, as Cl_2_ and HCl, is present in high concentrations in gaseous emissions from geothermal areas [46]. Since its content in the soil was below the NAA detection limit, it can be suggested that *F. ochotensis* leaves mainly accumulate Cl from aerial sources [45].

Among the trace elements known as essential for all plants, Al, Br, Cl, Co, Cu, Fe, Mn, Rb, Ti, V, and Zn were determined in *F. ochotensis*. The mass fractions of all these elements, except Cu, were higher in the analyzed plants compared to the RP, which can be explained by the specificity of the conditions under which they grow. Trace element uptake in plants near geothermal areas is primarily driven by naturally high geogenic concentrations, low soil pH, and high temperatures [47]. The plants also accumulated Cd, As, Sb, Hg, and rare earth elements, which have no biological function and can even be toxic to plants. The high mass fractions of elements in *F. ochotensis* may indicate its capacity for hyperaccumulation. Plants growing in polluted environments have developed physiological and biochemical mechanisms to tolerate and detoxify high concentrations of PTEs. These strategies prevent cellular damage and maintain metabolic functions in stressful conditions [48].

**Figure 2:**
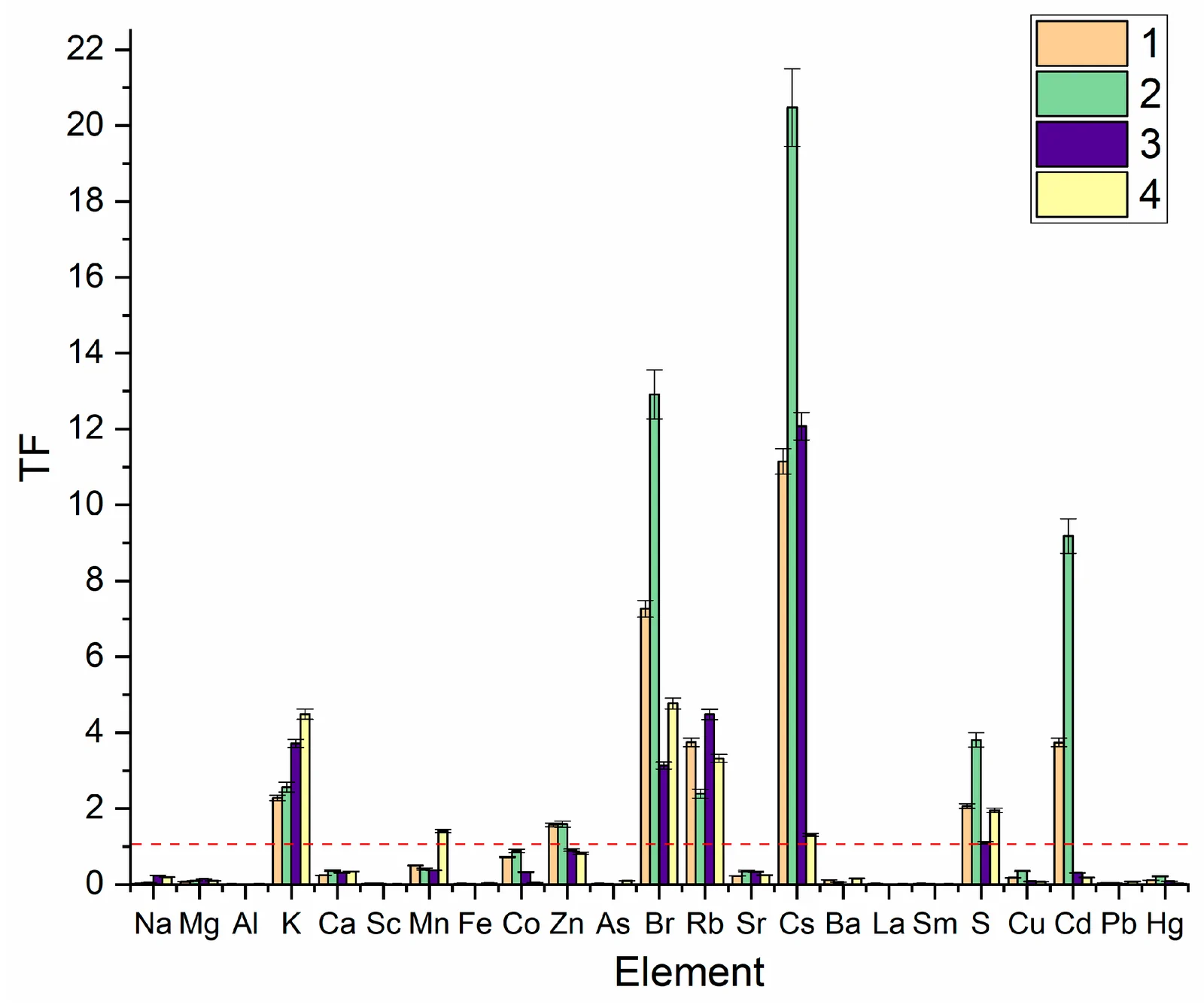
Transfer factor (TF) of elements from soil in *F. ochotensis* collected at hot springs of Kamchatka Peninsula (error bars indicate standard deviation).

Since plants in geothermal zones take up these elements either through root adsorption from hydrothermally altered soils or via foliar absorption from geothermal gases, transfer factor (TF) values were calculated (Figure 2). TFs below unity were obtained for Na, Mg, Al, Ca, Sc, Ti, V, Cr, Mn, Fe, Zn (sites 2 and 4), Co, As, Sr, Sb, Ba, La, Sm, Tb, Au, Th, Cu, Pb, Cd (sites 3 and 4), and Hg, indicating their low uptake by plants. Many metals, including Fe, Cr, Cu, Co, Pb, Ba, Sr, Th, and rare earth elements, have low mobility, which limits their accumulation in plants [37]. At the same time, plants can develop defense mechanisms in response to high concentrations of PTEs. 

TFs higher than unity were obtained for K, Rb, Cs, Br, Zn (sites 1 and 2), S, and Cd (sites 1 and 2). The alkali elements K, Rb, and Cs are among the most mobile elements in aquifer fluids, which facilitates their uptake by plants. In geothermal areas, bromine accumulates in surrounding soils and sediments as highly soluble, mobile bromide salts, which can be passively transported with water in plants [29,49]. Bromine and Cl can form strong complexes with Cd, increasing its phytoavailability in plants [45]. Accumulation of elements with TF < 1 can be attributed to aerial sources, since water-soluble gases and particulate matter may be incorporated into aerosol particles emitted with hot water in geothermal zones [50].

## 3. Materials and Methods

### 3.1. Sampling Area and Samples Collection

**Figure 3:**
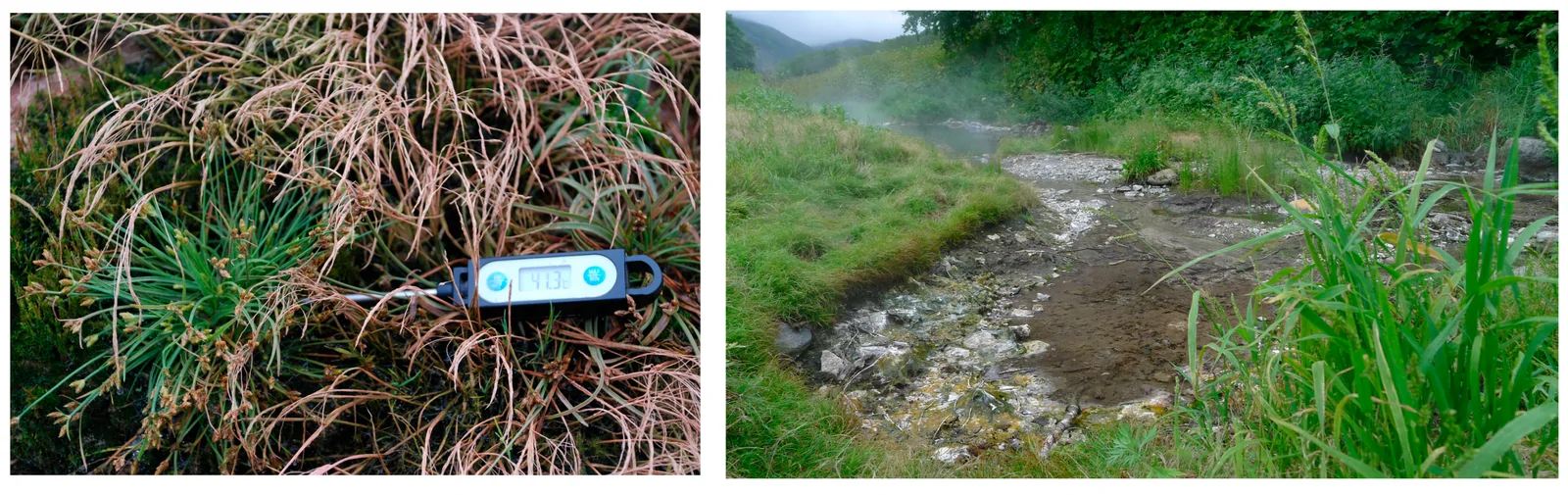
(**left**) *Fimbristylis ochotensis* and (**right**) *Fimbristylis ochotensis* at Malye Bannye Klyuchi of Kamchatka Peninsula.

**Figure 4:**
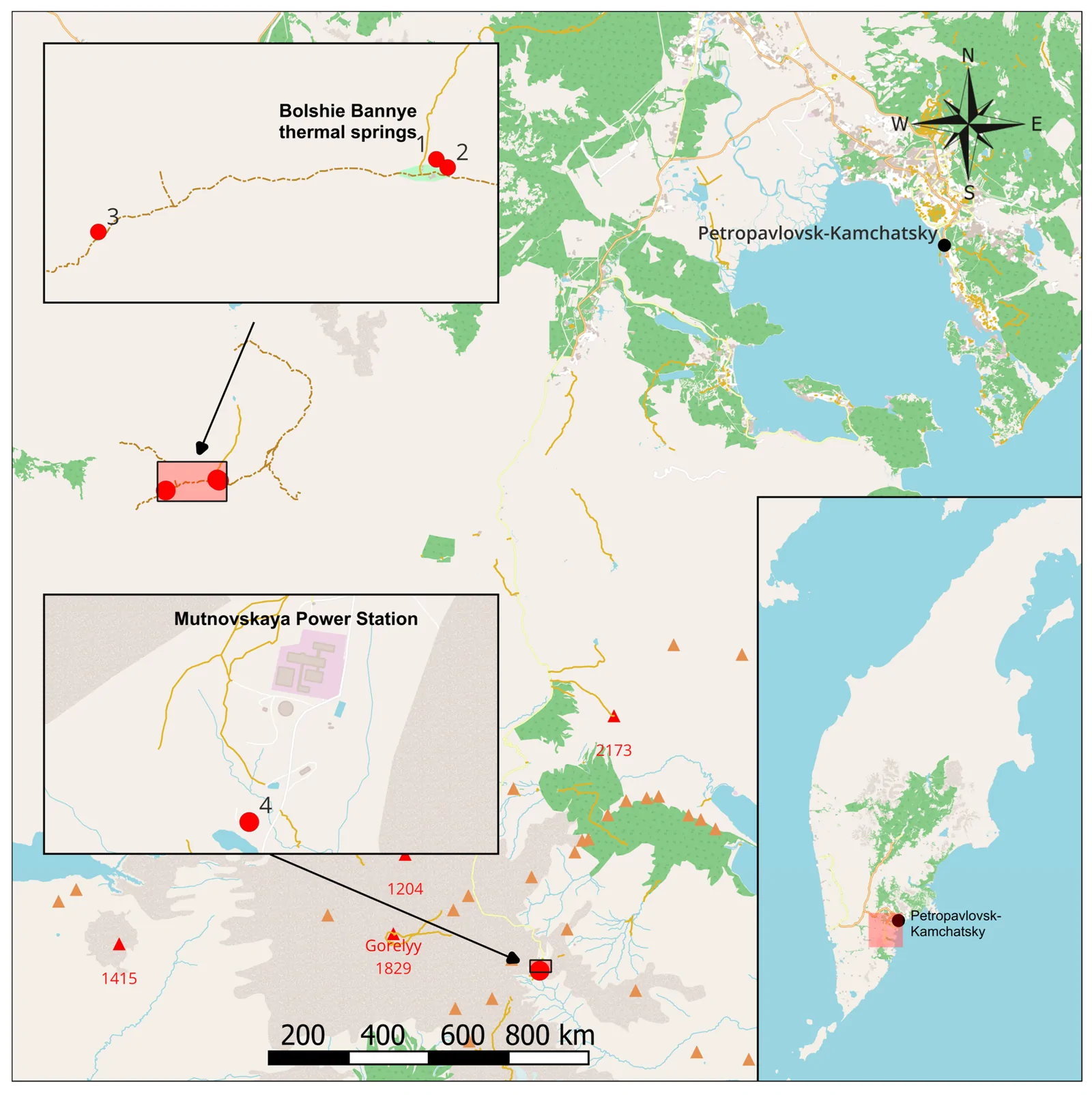
The map of *Fimbristylis ochotensis* sampling at hot springs of Kamchatka Peninsula.

Plant (Figure 3) and associated soil samples of *F. ochotensis* were collected at three hot springs on the Kamchatka Peninsula: Bolshie Bannye Klyuchi, Malye Bannye Klyuchi, and Dachnye Hot Springs (Figure 4).

Bolshie Bannye Klyuchi thermal field is located on the floodplain terrace of the left bank of the Bannaya River. It comprises 24 separate groups of springs, covering a total thermal area of approximately 1.5 km^2^. The site features more than 550 thermal outlets, including boiling, bubbling, and gushing vents—some displaying geyser-like behavior—along with concentrated and, less commonly, dispersed discharge points, as well as numerous mud pots. Chemically, the waters are siliceous, superheated, and low in mineralization (total mineralization 0.7–1.4 g/L), with a hydrocarbonate-chloride-sulfate sodium composition. They are neutral to slightly alkaline and contain dissolved carbonic and nitrogenous gases.

The Malye Bannye Klyuchi thermal springs are situated 5 km from the Bolshie Bannye Klyuchi field, in the upper reaches of the Bannaya River on its left bank, specifically along the right bank of the Malyi Klyuch River. The visible hot-water outlets, with temperatures reaching up to 79 °C, are concentrated within a compact area of approximately 36 m^2^. These discharges give rise to a warm stream that flows from the site.

Dachnye Hot Springs are located 1 km east of the pass between the Dvugorba and Skalistaya Mountains, 9 km north of Mutnovsky Volcano, in close proximity to the Mutnovskaya Geothermal Power Plant. Like the Severo-Mutnovsky Thermal Springs, they are more characterized by gas-steam jets than hot water springs. The chemical composition of the steam-gas jet condensate is sulfate-calcium-hydrocarbonate-sodium, with low total mineralization ranging from 0.12 to 0.7 g/L [51].

*F. ochotensis* (stems and leaves) and corresponding soil samples (ten independent biological specimens of each sample type) were collected from four sites in three hot springs zones. The temperature of the soil at Bolshie Bannye Klyuchi and Malye Bannye Klyuchi was around 40 °C, whereas at the other two sites it was near 70 °C. Soil samples were collected according to the Russian National Standard [52]. Before analysis, samples were dried at 105 °C to constant weight and homogenized. Samples for mercury determination were dried at 40 °C. 

### 3.2. Samples Elemental Analysis 

Each specimen was analyzed separately in three analytical replicates. Three analytical techniques—neutron activation analysis (NAA), inductively coupled plasma optical emission spectrometry (OES), and direct mercury analyzer—were used to determine the mass fractions of chemical elements in *F. ochotensis* plants and corresponding soil samples. 

Instrumental neutron activation analysis was performed at the Regata facility of the IBR-2 reactor in Dubna, Russia. For elements producing short-lived isotopes—namely Al, Mg, Cl, Ca, Ti, V, and Mn—plant material was irradiated for 3 min and soil samples for 1 min at a thermal neutron flux of 1.1 × 10^12^ n cm^−2^ s^−1^, followed by immediate gamma counting for 15 min. Elements yielding long-lived isotopes—including Na, K, Sc, Cr, Fe, Co, Ni, Zn, As, Br, Rb, Sr, Zr, Ga, Sb, Cs, Ba, La, Ce, Sm, Eu, Tb, Yb, Ta, Ho, Hf, W, Th, Au, Nd, Tm, and U—were determined after 4-day irradiations at a neutron flux of 1.2 × 10^11^ cm^−2^ s^−1^. Gamma spectra of induced activity were recorded after 4 and 20 days using Canberra HPGe detectors (efficiency 70–100%, resolution 1.8–2.0 keV at the 1332 keV ^60^Co total absorption peak). Spectral data were processed with Genie 2000 software, and mass fraction calculations were carried out using software developed at the Joint Institute for Nuclear Research (JINR) [53,54].

The mass fractions of Cd, Cu, S, and Pb were determined using a PlasmaQuant 9000 Elite (Analytik Jena, Jena, Germany). Prior to analysis, samples were digested in a Mars microwave digestion system (CEM, Matthews, NC, USA). Further methodological details are provided in Yushin et al. [55] and Zinicovscaia et al. [56]. Total mercury content was measured using a direct mercury analyzer (Milestone DMA-80evo, Milestone Srl, Milan, Italy). All measurements were conducted in triplicate.

Analytical accuracy was ensured through the analysis of certified reference materials (CRMs), including Oriental Basma Tobacco Leaves (INCT-OBTL-5) and IAEA-458 (sediments) for ICP-OES and direct mercury analyzer. Quality control of NAA measurements was ensured by irradiation of a set of CRMs: National Institute for Standard and Technology (NIST) 2709a (San Joaquin Soil), NIST 2709 (San Joaquin Soil), NIST 1515 (Apple Leaves), NIST 1547 (Peach Leaves), NIST 2710 (Montana Soil), NIST 1633b (Bituminous Coal Fly Ash), BCR-667 (Estuarine Sediment), and CTA-FFA-1 (Fine Fly Ash). Quality control results and methods detection limits can be found in previously published papers [57].

The pH of the soil was determined using Ohaus Starter 3100 pH meter (Ohaus, MA, USA) pH-meter according to procedure described in [58].

### 3.3. Data Evaluation

To assess the level of soil enrichment in studied elements Enrichment factor was calculated according to Equation (1):

(1) $$EF=\frac{C{\cdot Sc}_{b}}{C_{b}\cdot Sc}$$

where C and Sc represent the mass fractions of element of interest and Sc, respectively, in the analyzed soils, in mg kg^−1^, while Cb and Scb denote their corresponding background mass fractions, in mg kg^−1^. Scandium was selected as reference element [27]. The EF values were interpreted according to the following classification: EF < 2—minimal enrichment (negligible pollution), 2 ≤ EF < 5 moderate enrichment, 5 ≤ EF < 20 significant enrichment, 20 ≤ EF < 40 strong enrichment, EF ≥ 40 extreme enrichment [59].

Soil–plant transfer factor (TF) was calculated to assess elements uptake by plants (Equation (2)):

(2) $$TF=\frac{C_{leaves}}{C_{soil}}$$

where C_leaves_ is element’s mass fraction in the leaves, mg kg^−1^ and C_soil_ is the total mass fraction of the same element in the soil, mg kg^−1^. TF values ≥ 1 indicate high elements uptake from soil to plants [60].

### 3.4. Statistical Analysis

Microsoft Excel 2010 was used to collect the data and the descriptive statistics. All data are presented as the means ± standard deviations. Origin was applied for data visualization. Statistical analyses were performed using the individual biological specimens as the experimental units. Differences in elemental mass fractions among the four sampling sites were assessed separately for plant and soil samples using the Kruskal–Wallis test. When the overall test was significant (*p* < 0.05), Dunn’s post hoc test with Holm adjustment for multiple comparisons was applied to identify significant differences between individual pairs of sites.

## 4. Conclusions

This study provides the first comprehensive dataset on the elemental composition of the thermophyte plant *Fimbristylis ochotensis* of Kamchatka origin and its associated soils. Through the combined application of INAA, ICP-OES, and direct mercury analysis, 43 elements were quantified in soils and 31 in plant tissues, revealing substantial site-specific variation in elemental profiles. While the mass fractions of most elements were higher in the soil, the plant exhibited selective accumulation of Zn, Br, Rb, Cs, and Cd, with transfer factors exceeding unity. This study advances our understanding of the element bioaccumulation capacity and ecological adaptation of this obligate thermophyte and may serve as a basis for further investigations. The observed accumulation and transfer of elements suggest that *F. ochotensis* may have potential for phytoremediation of acidic, element-enriched soils; however, further studies are required to validate its phytoremediation potential under controlled and field conditions.

## Figures and Tables

**Table 1:** The mass fractions of elements (mg/kg) in soils collected at geothermal fields on the Kamchatka Peninsula along with upper continental crust values [27].

Element	1	2	3	4	UCC
Na	24,300 ± 500	26,200 ± 500	13,440 ± 270	11,260 ± 230	24,259
Mg	19,700 ± 1500	15,400 ± 1300	10,000 ± 900	14,600 ± 1200	14,957
Al	69,000 ± 3000	63,400 ± 2900	32,700 ± 1500	48,400 ± 2200	81,505
K	14,000 ± 600	16,400 ± 600	9300 ± 500	8300 ± 400	23,244
Ca	17,200 ± 1300	13,700 ± 1000	15,100 ± 1100	11,100 ± 900	25,568
Sc	12.8 ± 0.5	6.5 ± 0.2	8.9 ± 0.4	19 ± 0.8	14
Ti	3190 ± 230	2250 ± 210	2110 ± 160	5760 ± 280	3837
V	79 ± 2.8	41 ± 1.6	86 ± 2.7	99 ± 3	97
Cr	26 ± 1.7	11.1 ± 1.2	105 ± 4	34.2 ± 1.7	92
Mn	970 ± 50	2030 ± 90	720 ± 30	468 ± 22	774
Fe	31,000 ± 600	19,000 ± 400	99,100 ± 2000	22,000 ± 5000	39,176
Co	8.4 ± 0.2	8.5 ± 0.2	22.8 ± 0.4	7.6 ± 0.1	17
Ni	nd	nd	35 ± 2.4	nd	47
Zn	111 ± 4	49 ± 2.1	78 ± 3	42 ± 2.1	67
Ga	nd	nd	nd	14.6 ± 1.9	17.5
As	17.6 ± 0.7	42 ± 1.6	463 ± 17	10.5 ± 0.4	4.8
Br	37 ± 1.6	20 ± 0.9	62 ± 2.7	7.8 ± 0.3	1.6
Rb	45 ± 1.2	40 ± 1.2	21 ± 0.7	17.5 ± 0.5	84
Sr	234 ± 19	220 ± 15	237 ± 18	177 ± 12	320
Zr	107 ± 7	75 ± 5	98 ± 4	143 ± 8	193
Sb	1.6 ± 0.07	1.5 ± 0.05	22 ± 0.8	1.6 ± 0.07	0.4
Cs	12.7 ± 0.5	8.1 ± 0.3	14.5 ± 0.6	1.8 ± 0.07	4.9
Ba	570 ± 30	650 ± 30	250 ± 14	590 ± 30	628
La	12.5 ± 0.4	11.2 ± 0.4	6.9 ± 0.2	13.6 ± 0.4	31
Ce	25 ± 1.1	22 ± 0.9	17 ± 0.8	31 ± 1.2	63
Nd	14.3 ± 2	12 ± 1.6	6.5 ± 0.6	15.6 ± 2.1	27
Sm	2.6 ± 0.1	1.6 ± 0.9	2.0 ± 0.1	4.7 ± 0.2	4.7
Eu	0.7 ± 0.05	0.5 ± 0.004	0.6 ± 0.005	1.0 ± 0.07	1
Tb	0.42 ± 0.01	0.23 ± 0.01	0.30 ± 0.01	0.7 ± 0.03	0.7
Ho	0.75 ± 0.08	0.43 ± 0.05	0.6 ± 0.09	1.1 ± 0.1	0.83
Tm	0.3 ± 0.03	nd	0.2 ± 0.02	0.4 ± 0.04	0.3
Yb	2.2 ± 0.1	1.2 ± 0.1	1.5 ± 0.1	2.9 ± 0.2	2
Hf	3.45 ± 0.1	2.2 ± 0.1	1.9 ± 0.1	3.8 ± 0.2	5.3
Ta	0.3 ± 0.009	0.26 ± 0.008	0.13 ± 0.007	0.28 ± 0.009	0.9
W	3.6 ± 0.4	7.9 ± 0.9	52 ± 6	1.36 ± 0.1	1.9
Au	nd	nd	nd	0.18 ± 0.01	1.5
Th	3.4 ± 0.09	2.9 ± 0.07	1.5 ± 0.04	2 ± 0.05	10.5
U	1.3 ± 0.05	1.1 ± 0.03	0.7 ± 0.04	0.7 ± 0.03	2.7
S *	1265 ± 12	727 ± 2	3370 ± 50	6158 ± 29	621
Cu *	45 ± 0.2	16.9 ± 0.1	122 ± 1.1	31.7 ± 0.2	28
Cd *	0.26 ± 0.001	0.19 ± 0.002	0.6 ± 0.005	0.24 ± 0.002	0.09
Pb *	39 ± 0.7	27 ± 0.1	21 ± 0.7	11.8 ± 0.1	17
Hg **	0.25 ± 0.002	0.09 ± 0.001	0.21 ± 0.01	3.6 ± 0.02	0.05
pH	4.08	4.41	4.75	3.66	

*—determined by ICP-OES, **—determined by direct mercury analyzer.

**Table 2:** The mass fractions of elements (mg/kg) in *Fimbristylis ochotensis* plants collected at geothermal fields on the Kamchatka Peninsula along with “reference plant” (RP) values [40] (*—determined by ICP-OES, **—determined by direct mercury analyzer).

Element	1	2	3	4	RP
Na	762 ± 17	1217 ± 26	319 ± 66	2201 ± 45	150
Mg	1395 ± 313	1448 ± 325	1468 ± 320	1385 ± 310	2000
Al	1252 ± 58	705 ± 33	342 ± 16	1003 ± 46	80
Cl	14,153 ± 1110	21,714 ± 1700	16,585 ± 1300	13,220 ± 1040	2000
K	31,893 ± 840	42,023 ± 1090	34,579 ± 90	37,233 ± 960	19,000
Ca	4177 ± 180	4995 ± 220	4697 ± 200	3836 ± 170	10,000
Sc	0.32 ± 0.01	0.16 ± 0.01	nd	0.35 ± 0.01	0.02
Ti	nd	nd	nd	108 + 15	5
V	1.27 ± 0.07	nd	nd	1.29 ± 0.06	0.5
Cr	nd	nd	nd	7.35 ± 0.5	1.5
Mn	480 ± 26	825 ± 44	268 ± 14	662 ± 36	200
Fe	858 ± 34	433 ± 27	399 ± 26	913 ± 28	150
Co	6.1 ± 0.1	7.2 ± 0.2	7.4 ± 0.2	0.4 ± 0.02	0.2
Zn	174 ± 5.6	78 ± 2.7	70 ± 2.5	35 ± 1.3	50
As	0.45 ± 0.05	0.7 ± 0.07	3.6 ± 0.3	0.1 ± 0.07	0.1
Br	270 ± 11	260 ± 10	194 ± 8	37 ± 1	4
Rb	167 ± 5	97 ± 3	94 ± 4	58 ± 2	50
Sr	53 ± 5.3	78 ± 6.3	79 ± 6.6	43 ± 2.8	50
Sb	nd	nd	0.07 ± 0.001	0.07 ± 0.003	0.1
Cs	142 ± 6	166 ± 6	175 ± 7	2.4 ± 0.1	0.2
Ba	68 ± 4.3	41 ± 3.2	nd	96 ± 4.5	40
La	0.32 ± 0.04	nd	nd	0.29 ± 0.02	0.2
Sm	0.08 ± 0.005	0.03 ± 0.003	0.02 ± 0.002	0.09 ± 0.005	0.04
Tb	nd	nd	nd	0.01 ± 0.001	0.008
Au	nd	nd	nd	0.003 ± 0.0003	0.001
Th	nd	nd	nd	0.04 ± 0.002	0.005
S *	2610 ± 30	2767 ± 27	3690 ± 40	12,030 ± 30	3000
Cu *	8.1 ± 0.07	6.0 ± 0.02	9.8 ± 0.1	2.2 ± 0.08	10
Cd *	0.96 ± 0.003	1.79 ± 0.01	0.19 ± 0.005	0.05 ± 0.004	0.05
Pb *	1.5 ± 0.06	1.14 ± 0.17	0.47 ± 0.06	0.9 ± 0.01	1
Hg **	0.03 ± 0.001	0.02 ± 0.0001	0.02 ± 0.0001	0.1 ± 0.004	0.1

## Data Availability

All results are presented in the manuscript.
